# Saline versus balanced crystalloids for intravenous fluid therapy in the emergency department: study protocol for a cluster-randomized, multiple-crossover trial

**DOI:** 10.1186/s13063-017-1923-6

**Published:** 2017-04-13

**Authors:** Wesley H. Self, Matthew W. Semler, Jonathan P. Wanderer, Jesse M. Ehrenfeld, Daniel W. Byrne, Li Wang, Leanne Atchison, Matthew Felbinger, Ian D. Jones, Stephan Russ, Andrew D. Shaw, Gordon R. Bernard, Todd W. Rice

**Affiliations:** 1 Department of Emergency Medicine, 1313 21st Avenue South, 703 Oxford House, Nashville, TN 37220 USA; 2 Division of Allergy, Pulmonary and Critical Care Medicine, Nashville, TN USA; 3 Department of Anesthesiology, Nashville, TN USA; 4 Department of Biomedical Informatics, Nashville, TN USA; 5 Department of Biostatistics, Nashville, TN USA; 6 Department of Pharmaceutical Services, All at Vanderbilt University Medical Center, Nashville, TN USA

**Keywords:** Intravenous fluids, Crystalloid, Saline, Renal failure, Pragmatic trial, Emergency department

## Abstract

**Background:**

Prior studies in critically ill patients suggest the supra-physiologic chloride concentration of 0.9% (“normal”) saline may be associated with higher risk of renal failure and death compared to physiologically balanced crystalloids. However, the comparative effects of 0.9% saline and balanced fluids are largely unexamined among patients outside the intensive care unit, who represent the vast majority of patients treated with intravenous fluids.

**Methods/design:**

This study, entitled Saline Against Lactated Ringer’s or Plasma-Lyte in the Emergency Department (SALT-ED), is a pragmatic, cluster, multiple-crossover trial at a single institution evaluating clinical outcomes of adults treated with 0.9% saline versus balanced crystalloids for intravenous fluid resuscitation in the emergency department. All adults treated in the study emergency department receiving at least 500 mL of isotonic crystalloid solution during usual clinical care and subsequently hospitalized outside the intensive care unit are included. Treatment allocation of 0.9% saline versus balanced crystalloids is assigned by calendar month, with study patients treated during the same month assigned to the same fluid type. The first month (January 2016) was randomly assigned to balanced crystalloids, with each subsequent month alternating between 0.9% saline and balanced crystalloids. For balanced crystalloid treatment, clinicians can choose either Lactated Ringer’s or Plasma-Lyte A©. The study period is set at 16 months, which will result in an anticipated estimated sample size of 15,000 patients. The primary outcome is hospital-free days to day 28, defined as the number of days alive and out of the hospital from the index emergency department visit until 28 days later. Major secondary outcomes include proportion of patients who develop acute kidney injury by creatinine measurements; major adverse kidney events by hospital discharge or day 30 (MAKE30), which is a composite outcome of death, new renal replacement therapy, and persistent creatinine elevation >200% of baseline; and in-hospital mortality.

**Discussion:**

This ongoing pragmatic trial will provide the most comprehensive evaluation to date of clinical outcomes associated with 0.9% saline compared to physiologically balanced fluids in patients outside the intensive care unit.

**Trial registration:**

ClinicalTrials.gov, NCT02614040. Registered on 18 November 2015.

**Electronic supplementary material:**

The online version of this article (doi:10.1186/s13063-017-1923-6) contains supplementary material, which is available to authorized users.

## Background

Administration of intravenous isotonic fluid is one of the most common therapies in hospitals today [1]. However, the optimal content for these intravenous fluids remains unknown [1–3]. In the USA, 0.9% (“normal”) saline is the most commonly used isotonic fluid, with more than 200 million liters administered annually [1]. The chloride concentration of 0.9% saline (154 mEq/L) is higher than that of human plasma (94–111 mEq/L), which causes a hyperchloremic metabolic acidosis when 0.9% saline is used as a resuscitation fluid [1, 2]. Accumulating evidence from critically ill patients suggests that this supra-physiologic chloride concentration may also lead to kidney injury and impair a patient’s ability to recover from severe illness [4–9]. Chloride concentrations in physiologically balanced crystalloids, such as Lactated Ringer’s (109 mEq/L) and Plasma-Lyte© (98 mEq/L), are similar to that of human plasma, potentially making them safer alternatives for isotonic fluid resuscitation [1, 2].

Ongoing trials, including the Isotonic Solutions and Major Adverse Renal Events Trial (SMART) (NCT02444988) and the Plasma-Lyte 148 versus Saline Trial (PLUS) (NCT02721654), are being conducted to more definitively evaluate the comparative effects of 0.9% saline and balanced crystalloids on kidney function and mortality in critically ill patients in the intensive care unit (ICU). While critically ill ICU patients are likely the most vulnerable to the potential detrimental effects of 0.9% saline, most patients treated with isotonic fluids are not critically ill [1, 10, 11]. Therefore, quantifying the clinical implications of 0.9% saline therapy on patients with less severe illness outside the ICU is essential. Due to the large number of patients outside the ICU exposed to isotonic fluids, even small risk differences between 0.9% saline and balanced crystalloids for individual patients may translate into substantial differences on a population level. The comparative effects of 0.9% saline and balanced fluids on patients without critical illness are largely unstudied. Therefore, the current trial was designed to evaluate short-term clinical outcomes of adults treated with 0.9% saline versus balanced crystalloids in the emergency department (ED) and subsequently hospitalized outside the ICU. We hypothesize that treatment with balanced crystalloids compared to 0.9% saline will result in more hospital-free days, defined as days alive and out of the hospital between the index ED visit and 28 days later. Results of this study combined with other ongoing trials focusing on ICU patients will provide a comprehensive evaluation of the comparative clinical effects of 0.9% saline and balanced crystalloids across the full spectrum of diseases typical for hospitalized adults.

## Methods/design

### Design

This study, entitled Saline Against Lactated Ringer’s or Plasma-Lyte in the Emergency Department (SALT-ED), is a prospective, pragmatic, single-center, unblinded, cluster, multiple-crossover trial [12, 13] comparing the clinical outcomes of adults hospitalized after treatment with 0.9% saline versus physiologically balanced crystalloids in the ED. Consistent with the concept of a pragmatic clinical trial [14], eligibility criteria are broad, the sample size is large, and study procedures are embedded into routine care and executed by clinical personnel. The trial was approved by the Vanderbilt University Medical Center Institutional Review Board (IRB) with waiver of informed consent as a comparison of two interventions that are routinely prescribed during usual clinical care (IRB 151769). This is an investigator-initiated trial that was registered with ClinicalTrials.gov (NCT02614040) prior to initiation of patient enrollment, with progress and safety monitored by an independent Data and Safety Monitoring Board (DSMB). The Vanderbilt Institute for Clinical and Translational Research provided funding through a Clinical and Translational Science Award (CTSA) from the National Center for Advancing Translational Sciences (UL1 TR000445). Funding sources have no role in the design, conduct, or interpretation of the trial. The trial protocol was developed according to the Standardized Protocol Items: Recommendations for Interventional Trials (SPIRIT) guidelines (see Additional file 1 and Additional file 2: Figure S1) [15].

### Population

**Fig. 1:**
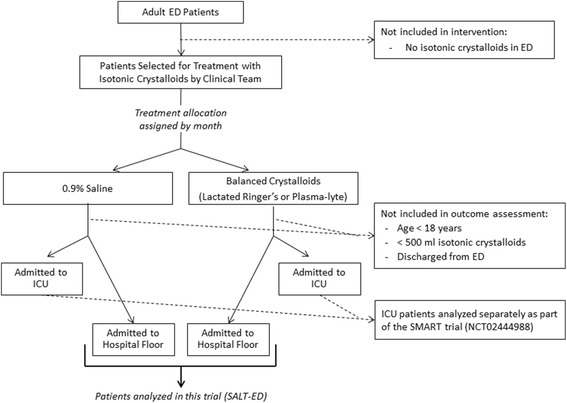
Flow diagram of patient participation

The trial is being conducted at Vanderbilt University Medical Center, a tertiary care academic hospital in Nashville, Tennessee, USA, with approximately 75,000 adult ED visits per year. The study population includes adults (≥18 years old) treated with ≥500 mL of intravenous isotonic fluids in the ED and subsequently admitted to the acute care hospital outside the ICU (Fig. 1). Patients treated with <500 mL of fluids are not included, because this limited volume is unlikely to be given for resuscitation and 0.9% saline versus balanced crystalloids is unlikely to lead to important clinical differences at this low dose of exposure [10]. The study period is 01 January 2016 through 30 April 2017. Individual patients may contribute multiple hospitalizations to the trial if they meet eligibility criteria more than once during the study period.

If a treating clinician decides to administer isotonic fluids, the study protocol dictates the type of isotonic fluid administered in the ED. All other aspects of patient care are determined by treating clinicians independent of the study protocol, including whether to treat with fluids, the volume of fluid resuscitation, and admission decisions. Patients admitted from the ED to a non-ICU floor will be analyzed in this study. Patients admitted from the ED to an ICU will be analyzed in a complementary trial of critically ill ICU patients (SMART, NCT02444988).

### Treatment interventions

Two different types of isotonic crystalloids—0.9% saline versus physiologically balanced crystalloids—are being compared. Balanced crystalloids are characterized by a chloride concentration similar to that of human plasma and lower than 0.9% saline (Table 1). Lactated Ringer’s and Plasma-Lyte are the balanced crystalloids commonly available in the USA [1]. In this trial, treating clinicians can select either Lactated Ringer’s or Plasma-Lyte A for patients assigned to balanced crystalloids, because slight content differences lead some clinicians to prefer one over the other for particular patients. For example, some clinicians prefer Plasma-Lyte over Lactated Ringer’s for patients undergoing blood transfusions because it has been hypothesized that the calcium in Lactated Ringer’s may bind the citrate anticoagulant in packed red blood cells and increase the risk of microthrombi [1, 16]. Allowing clinicians to select either Lactated Ringer’s or Plasma-Lyte as balanced crystalloids improves clinician support for the trial and emulates clinical practice while maintaining relevant comparator groups consisting of chloride-rich fluids (0.9% saline) versus fluids with a chloride concentration similar to plasma (Lactated Ringer’s or Plasma-Lyte).

**Table 1:** Content of human plasma, 0.9% saline, Lactated Ringer’s, and Plasma-Lyte A

			Balanced crystalloids	
	Human plasma	0.9% saline	Lactated Ringer’s	Plasma-Lyte A©
Sodium (mEq/L)	135–145	154	130	140
Potassium (mEq/L)	4.5–5.0	0	4	5
Chloride (mEq/L)	94–111	154	109	98
Calcium (mEq/L)	2.2–2.6	0	2.7	0
Magnesium (mEq/L)	0.8–1.0	0	0	3
Bicarbonate (mEq/L)	23–27	0	0	0
Lactate (mEq/L)	1–2	0	28	0
Acetate (mEq/L)	0	0	0	27
Gluconate (mEq/L)	0	0	0	23

### Treatment allocation

**Fig. 2:**
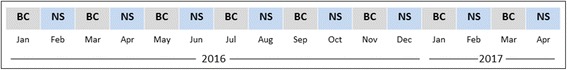
Schedule of treatment allocation for the study emergency department. *BC* balanced crystalloids, *NS* 0.9% (“normal”) saline

Treatment allocation is assigned by calendar month, with all patients in the study ED during the same month treated with the same type of isotonic fluid: 0.9% saline or balanced crystalloids. Clinicians and patients are not blinded to treatment allocation. The first study month (January 2016) was assigned by computer-generated simple randomization to balanced crystalloids. Treatment allocation then sequentially crosses over between balanced crystalloids and 0.9% saline each month, so that the trial will include 16 total months with 8 months each of balanced crystalloids and 0.9% saline (Fig. 2). This creates a sequential, cluster, multiple crossover trial design with one cluster (the study ED) and 16 periods (the 16 study months) [12, 13].

Adherence to study-assigned fluids is achieved by a multifaceted approach, including clinician education, electronic order entry advisors, and pharmacy supply. We have previously validated this approach to assignment of 0.9% saline versus balanced crystalloids, demonstrating high compliance with assigned treatment groups [10].

All ED clinicians, including physicians, nurse practitioners, pharmacists, and nurses, are informed about the intravenously (IV) administered fluid treatment assignment for each month in a continuous process of email reminders, announcements, and posted signs in the ED.

**Fig. 3:**
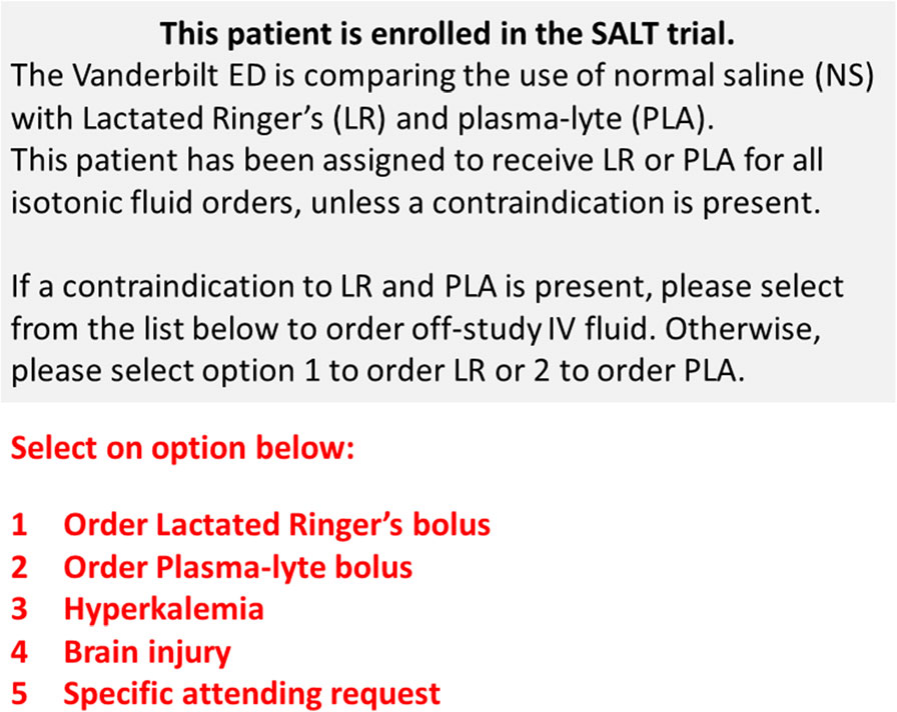
Electronic advisor embedded into the computerized provider order entry system alerting a clinician he/she has ordered 0.9% saline during a balanced fluid month. A similar electronic alert also informs clinicians when they have ordered balanced crystalloids during a 0.9% saline month

Electronic advisors embedded in the computerized provider order entry system encourage isotonic fluid selection consistent with the study protocol. If a provider orders isotonic fluid consistent with protocol (e.g., 0.9% saline during a 0.9% saline month), the orders are processed without interruption. If a provider orders isotonic fluids discordant with the protocol (e.g., 0.9% saline during a balanced crystalloids month), an electronic advisor appears alerting the provider about the ongoing trial and the assignment of this patient to a different type of isotonic fluid (Fig. 3). This electronic advisor gives the provider an option to order an off-protocol fluid type for specific reasons. Although no absolute contraindications exist for selecting 0.9% saline or balanced crystalloids as a resuscitation fluid, some clinicians prefer 0.9% saline for patients with hyperkalemia due to the higher potassium content in balanced crystalloids, and for patients with brain injury because the balanced crystalloids have lower tonicity. The advisor accepts hyperkalemia and brain injury as indications to administer 0.9% saline during months assigned to balanced crystalloids. Additionally, attending physicians can override the advisor any time they believe a specific fluid is needed for the safe treatment of an individual patient. Investigators evaluate the advisor overrides on a daily basis to monitor protocol compliance and identify if any providers frequently override the advisor.

The study ED is preferentially supplied by the hospital pharmacy each month with isotonic fluid assigned by the study protocol. This includes stocking treatment rooms and automated medicine dispensing cabinets with either 0.9% saline or Lactated Ringer’s and Plasma-Lyte, depending on the month. Upon special request from the treating clinician, alternative, off-protocol isotonic fluids are available.

### Data collection

Data are collected using queries of the electronic medical records and electronic data warehouse at Vanderbilt University Medical Center. We have previously validated these data collection techniques for relevant data points, including type and volume of fluids received, in-hospital death, new renal replacement therapy, persistent renal dysfunction by creatinine measurement, and hospital length of stay [17, 18]. Clinical providers are encouraged to report any potential adverse events from the trial to the investigators, who will report these findings to the governing IRB and DSMB.

### Outcomes

The primary outcome is hospital-free days to day 28, defined as the number of days alive and outside the hospital between the index ED visit and 28 days later. This outcome is a composite of in-hospital mortality and hospital length of stay, and has a range from 0 days (most severe outcome) to 28 days (least severe outcome). Patients who die in the hospital are assigned 0 hospital-free days to weigh death as the most severe outcome [19]. Patients who are hospitalized for ≥28 days are assigned 0 hospital-free days. Patients discharged prior to day 28 are assumed to survive to day 28 and are assigned [28 – hospital length of stay] hospital-free days.

All secondary outcomes are assessed until hospital day 28 or discharge, whichever occurs first, except where noted below. Secondary outcomes include: (1) proportion of patients who develop stage II or greater acute kidney injury by Kidney Disease Improving Global Outcomes (KDIGO) creatinine criteria [20]; (2) proportion of patients with a major adverse kidney event by hospital discharge or day 30 (MAKE30) [21], which is a composite outcome of death, new renal replacement therapy, or persistent creatinine elevation >200% of baseline; (3) in-hospital mortality during entire hospitalization; (4) hospital length of stay for the entire hospitalization; (5) ICU-free days (all “free-day” methodology matching that described for hospital-free days above); (6) ventilator-free days; (7) vasopressor-free days; (8) proportion of patients with new renal replacement therapy; (9) duration of new renal replacement therapy; (10) peak serum creatinine concentration; (11) change in serum creatinine concentration from baseline to peak; (12) proportion of patients with acidosis (serum bicarbonate <20 mmol/L) and alkalosis (serum bicarbonate >30 mmol/L); and (13) proportion of patients with hyperchloremia (serum chloride >110 mmol/L) and hypochloremia (serum chloride <90 mmol/L).

### Subgroups

In addition to the overall study population, primary and secondary outcomes will also be compared between the 0.9% saline and balanced crystalloid groups within several clinically important subpopulations, including: (1) admitting inpatient service (medicine, cardiology, surgery, trauma); (2) age ≥65 years; (3) chronic renal replacement therapy; (4) ED serum creatinine ≥1.5 mg/dL; (5) ED serum bicarbonate <20 mmol/L; (6) ED serum chloride >110 mmol/L; and (7) volume of crystalloid administered in the ED >2 liters.

### Statistical analysis

Analysis will be conducted using R version 3.2.0 (R Foundation for Statistical Computing, Vienna, Austria) and STATA version 14 (Stata Corp, College Station, TX, USA).

#### Primary analysis

The primary analysis will be an intention-to-treat analysis of eligible patients assigned to 0.9% saline versus balanced crystalloids based on the primary outcome of hospital-free days. In an unadjusted analysis, hospital-free days will be compared between study groups (0.9% saline versus balanced crystalloids) using the Wilcoxon rank-sum test. We will also construct a multivariable proportional odds model with the dependent variable of hospital-free days, and independent variables including study group and the following covariates: age, sex, race, admitting inpatient service (medicine, cardiology, surgery, trauma), and days elapsed since the initiation of the study on 01 January 2016.

#### Secondary analyses

Using the intention-to-treat population, secondary outcomes will be compared between study groups using the same approach described for the primary outcome. A per-protocol secondary analysis will also be performed in which eligible patients who were assigned to and received 0.9% saline will be compared to patients who were assigned to and received balanced crystalloids. Heterogeneity of treatment effect will be evaluated by comparing the primary outcome between study groups within the pre-specified subgroups above. These subgroup analyses will be conducted using interaction terms within the primary proportional odds model [22].

The comparative effects of 0.9% saline and balanced crystalloids may be modified by the volume of fluid received [10]. Therefore, we will also perform an analysis evaluating for this potential interaction by constructing a proportional odds regression model with the dependent variable as hospital-free days, and independent variables as assigned study group (0.9% saline versus balanced crystalloids), volume of crystalloids received in the ED, and the interaction term between study group and volume of crystalloids.

A two-sided *p* value < 0.05 will be considered statistically significant. Secondary analyses will be considered hypothesis generating, and no statistical corrections will be made for multiple comparisons.

#### Power calculation

The study duration has been set at 16 months, and the sample size will be the number of patients treated with isotonic intravenous fluids in the ED and admitted to a non-ICU hospital floor during that time. Sixteen months was chosen as the study duration to ensure numerous crossovers between 0.9% saline and balanced crystalloid months, enrollment throughout the academic and calendar year, adequate time for incorporation of study procedures into clinical care, a large sample size to balance baseline characteristics between groups, and adequate sample size to detect a 0.5-day difference in hospital-free days.

Based on historical data from the study ED, we anticipate a sample size of approximately 15,000 patients, with an even split between the 0.9% saline and balanced crystalloid groups. The anticipated mean for hospital-free days in the 0.9% saline group is 24 days with a standard deviation of 4 days. Based on these assumptions, 15,000 patients would provide greater than 90% power to detect a difference of 0.5 hospital-free days between groups with a type I error rate of 0.05.

#### Missing data

Data for this pragmatic clinical trial are available in the administrative data warehouse and electronic medical records at our institution. Based on prior work with these systems [10, 17, 18], we anticipate no missing data for intervention group assignment (0.9% saline versus balanced crystalloids) or the primary outcome (hospital-free days). Among the secondary outcomes, we anticipate some missing data for baseline creatinine levels, defined as a serum creatinine measurement during the one year prior to the index ED visit. In a pilot of 974 patients at our institution, 379 (39%) did not have a baseline creatinine measurement available [10]. Baseline creatinine values will be imputed for patients with missing values using a previously described equation [creatinine = 0.74 – 0.2 (if female) + 0.08 (if Black) + 0.003 × age (in years)] [23].

#### Interim analysis

As planned in the study protocol, an independent DSMB consisting of physician-scientists not involved in the trial reviewed data for patients enrolled during the first half of the study (January–August 2016). The DSMB recommended continuing enrollment until the planned stop date of 30 April 2017. Investigators were blinded to these data reviewed by the DSMB. The stopping boundary for efficacy during the DSMB review was defined as a difference in hospital-free days of at least 0.5 days with a *p* value < 0.001. Because even small differences between groups could be clinically meaningful for an intervention used as frequently as intravenous fluids, no stopping boundary for futility was defined. Due to the conservative Haybittle-Peto boundary (*p* < 0.001) used for the interim analysis, the significance level for the final analysis (*p* < 0.05) will not be adjusted [24].

## Discussion

Upon completion, the SALT-ED trial will provide the most comprehensive available data on the comparative clinical effects of 0.9% saline versus balanced crystalloids for fluid resuscitation of acutely ill patients in the ED. Given the accumulating evidence suggesting that the supra-physiologic chloride concentration of 0.9% saline may lead to adverse clinical manifestations [4–10], these data will be important for evaluating the safety of 0.9% saline as a resuscitation fluid for routine use. Results showing superior clinical outcomes in the balanced crystalloids group would provide compelling evidence that balanced solutions should be considered the preferred resuscitation fluid in most acutely ill patients. Null results or better clinical outcomes with 0.9% saline would help cement 0.9% saline as a first-line resuscitation fluid and encourage researchers and clinicians to move beyond the current debate about the optimal content of resuscitation fluids and focus on other aspects of acute care for improving patient outcomes.

While designing the SALT-ED trial, we considered the relative advantages and disadvantages of several designs, including individual patient randomization and blinding fluid type assignments. We selected an unblinded, pragmatic, cluster multiple-crossover trial to capture all isotonic fluids administered in the ED as part of the trial and to study the effects of 0.9% saline versus balanced crystalloids in a real-world, clinical environment. Clinical practice guidelines emphasize prompt initiation of fluid resuscitation for many illnesses in the ED [25]. Hence, resuscitation fluids are often administered within minutes of a patient reaching the ED and before electronic orders are entered, making it impractical for investigators to assign 0.9% saline versus balanced crystalloids after patient arrival in the ED and before initiation of fluid administration. Treatment allocation on an individual patient basis, such as with an individual randomized controlled trial, would likely lead to frequent crossover in the type of resuscitation fluids received by individual patients between the fluid type initiated by clinicians immediately after ED arrival and the fluid type assigned by the study at a later time. This type of unplanned crossover would bias results toward the null and severely jeopardize the trial [26]. Meanwhile, patient-level randomization with exclusion of patients who received isotonic fluids prior to randomization would likely lead to systematic exclusion of the most severely ill patients, who tend to receive fluids early in their ED course; these severely ill patients are likely to receive the greatest volume of isotonic fluid and are at greatest risk for adverse effects from 0.9% saline, making them essential to study.

Therefore, we selected clustered assignment of the intervention for the entire ED, alternating assignment based on calendar month. This enabled clinicians to immediately treat patients upon ED arrival with resuscitation fluids consistent with the study protocol. This simple design of assigning 0.9% saline versus balanced crystalloids was immediately accepted by ED clinicians and incorporated into routine care, facilitating high compliance with the study protocol and inclusion of the broadest range of patients in the trial. A design with numerous short periods and frequent crossovers was selected to minimize the risk of changes over time in the patient population and usual care confounding trial results [12, 26].

Potential threats to the validity of a trial with unblinded, clustered treatment allocation include biased outcome assessment due to known treatment allocation and carryover effects from one period to another [12]. To reduce the risk of biased outcome assessment, we selected objective outcomes with data collected directly from the electronic health records, including length of hospital stay, death, initiation of renal replacement therapy, and creatinine measurements. The risk of carryover between 0.9% saline and balanced crystalloid periods is low, despite no washout time between periods, because the simple switch from one fluid type to another at midnight on the first day of each month is unlikely to affect other aspects of care delivered in the ED. There are no changes to clinical care implemented in one period that are not completely reversed by changing the fluid type at the beginning of the next period.

### Trial status

In summary, the SALT-ED trial is an ongoing, pragmatic, cluster, multiple-crossover trial that will provide clinical outcome data for adults treated with 0.9% saline versus balanced crystalloids in the ED and subsequently hospitalized outside the ICU. The first patient was enrolled on 01 January 2016, and enrollment is scheduled for completion on 30 April 2017.

## Additional files

Additional file 1: SPIRIT checklist. (DOCX 61 kb)

Additional file 2: Figure S1. Schedule of enrollment, interventions, and assessments for the Saline Against Lactated Ringer’s or Plasma-Lyte in the Emergency Department (SALT-ED) trial. (DOCX 17 kb)

## Abbreviations

CTSA: Clinical and Translational Science Award
dL: Deciliter
DSMB: Data and Safety Monitoring Board
ED: Emergency department
ICU: Intensive care unit
IRB: Institutional Review Board
KDIGO: Kidney Disease Improving Global Outcomes
L: liter
MAKE30: Major Adverse Kidney Event by Day 30
mEq: Milliequivalent
mg: Milligram
mL: Milliliter
PLUS: Plasma-Lyte 148 versus Saline Trial
SALT-ED: Saline Against Lactated Ringer’s or Plasma-Lyte in the Emergency Department
SMART: Isotonic Solutions and Major Adverse Renal Events Trial
