# Supplementary material for: Saline versus balanced crystalloids for intravenous fluid therapy in the emergency department: study protocol for a cluster-randomized, multiple-crossover trial
Source: Trials. 2017 Apr 13;18:178. doi: 10.1186/s13063-017-1923-6 (PMC5390477; doi:10.1186/s13063-017-1923-6)
Supplement: Supplementary file 2 — Schedule of enrollment, interventions, and assessments for the Saline Against Lactated Ringer’s or Plasma-Lyte in the Emergency Department (SALT-ED) trial. (DOCX 17 kb) [file 13063_2017_1923_MOESM2_ESM.docx]

Figure S1. Schedule of enrolment, interventions, and assessments for the Saline Against Lactated Ringer’s or Plasmalyte in the Emergency Department (SALT-ED) trial.

|  |  | **STUDY PERIOD** | | |
| --- | --- | --- | --- | --- |
|  |  | **Post-allocation** | | |
| **TIMEPOINT** | **t_0_ (ED)** | ***t_1_ (earlier of day 28 or discharge)*** | ***t_2_ (earlier of day 30 or discharge)*** | ***t_3_ (hospital discharge)*** |
| **ENROLMENT:** |  |  |  |  |
| **Enrolment** | X |  |  |  |
| **Allocation** | X |  |  |  |
| **INTERVENTIONS:** |  |  |  |  |
| ***0.9 saline vs balanced crystalloid*** | X |  |  |  |
| **ASSESSMENTS:** |  |  |  |  |
| ***Baseline characteristics*** | X |  |  |  |
| ***Hospital-free days*** |  | X |  |  |
| ***Acute kidney injury*** |  | X |  |  |
| ***MAKE30*** |  |  | X |  |
| ***Mortality*** |  |  |  | X |
| ***Length of Stay*** |  |  |  | X |
| ***ICU-free days*** |  | X |  |  |
| ***Ventilator-free days*** |  | X |  |  |
| ***Vasopressor-free days*** |  | X |  |  |
| ***Renal replacement therapy*** |  | X |  |  |
| ***Creatinine values*** |  | X |  |  |
| ***Bicarbonate values*** |  | X |  |  |
| ***Chloride values*** |  | X |  |  |
